# A Combination of Caffeine, TeaCrine, and Dynamine Improves the Neurophysiological and Performance Measures of Electronic (E)-Gamers

**DOI:** 10.7759/cureus.44254

**Published:** 2023-08-28

**Authors:** Cassandra Evans, Jose Antonio, Amani Khan, Alexandra Vanderkley, Maria Berrocales, Jose Rojas, Samir Sakaria, Joseph Petruzzelli, Juan Carlos Santana, Jason Curtis, Tony Ricci, Jaime L Tartar

**Affiliations:** 1 Health Care Sciences, Nova Southeastern University, Fort Lauderdale, USA; 2 Human and Sport Performance, Rocky Mountain University of Health Professions, Provo, USA; 3 Psychology and Neuroscience, Nova Southeastern University, Fort Lauderdale, USA; 4 Health Sciences, Nova Southeastern University, Fort Lauderdale, USA; 5 Sports Science, Institute of Human Performance, Boca Raton, USA; 6 Exercise Science, Keiser University, West Palm Beach, USA; 7 Exercise and Sport Science, Nova Southeastern University, Fort Lauderdale, USA

**Keywords:** teacrine, egamer, dynamine, caffeine, attention

## Abstract

Introduction: Video games require precise motor skills, quick reaction times, and cognitive engagement. The tremendous growth of the electronic (e)-gaming industry has increased the demands for cognitive supplements (e.g., nootropics) to help e-athletes gain a competitive edge. The primary aim of this study was to evaluate the effects of combined caffeine + TeaCrine + Dynamine measures of neurophysiological and first-person shooter game performance in e-gamers.

Methods: Using a randomized double-blinded, crossover design, we assessed the effects of an acute, single-dose treatment of caffeine (200 mg) vs. caffeine (200 mg) + TeaCrine (10 mg) + Dynamine (50 mg) (CTD) vs. Ppacebo (maltodextrin). Each participant was tested under all three conditions one week apart. Baseline and post-dose measures were tested one hour apart. Participants [n = 49 male (24.4 ±, 4.5 yr)] were amateur e-gamers who play a first-person video game for at least 10 hours/week. Gaming performance was assessed through a series of first-person shooter training games through AIMLAB (State Space Labs, Inc., New York, USA). These included Reflex Shot (RS) standard, speed, and precision. The neurophysiological activity was captured while participants played three games through a single-channel EEG.

Results: In the standard game, the caffeine and the CTD conditions shot significantly more targets relative to the placebo, and both caffeine and the CTD condition had significantly greater targets post-dose compared to pre-dose. However, both the placebo and caffeine conditions had significantly slower reaction times post-dose compared to pre-dose. In the speed game, both the caffeine and placebo conditions shot a significantly greater number of targets, while the placebo and caffeine conditions had significantly more shots post-dose compared to pre-dose. Only the CTD condition had a significant increase in total kills post-dose compared to pre-dose. In the precision game, only the CTD condition significantly improved the number of kills per second post-dose, while only the caffeine condition had more shots post-dose. EEG data collected concomitantly with game playing showed that the CTD condition resulted in significantly lower alpha power compared to the placebo condition. The CTD group also showed increased theta activity post-dose during game playing compared to both the placebo caffeine conditions.

Conclusion: CTD appears to improve overall shooting gaming performance and neurophysiological measures of cognitive activity compared to caffeine and placebo. Collectively, these findings suggest that CTD assists with speed-accuracy tradeoffs where caffeine-only can lead to erratic play; thus, CTD may be particularly beneficial for shooting precision. The EEG data support this notion since the CTD exhibited lower alpha power suggesting increased cognitive flexibility and arousal and higher theta power suggesting greater cognitive control and decision-making under pressure.

## Introduction

The popularity of electronic (e)-games is growing exponentially [1,2]. There are different classifications of e-sports. First-person shooter games are a genre of games in which the player is participating in weapon-based combat from a first-person perspective [1]. As e-sports gain global recognition in the sports industry, there is a need for supplements to help gain a competitive edge. Similar to other skill-based sports, supplements geared toward enhancing cognitive performance are best suited for e-gamers. Video games require fine motor skills, fast reaction times, sustained focus, and eye-hand coordination.

The effects of caffeine as an ergogenic aid for sports performance and skill-based sports are well established [3-5]. Caffeine exerts its effects on the adenosine receptors, increasing the release of neurotransmitters. This leads to an increase in alertness and enhanced cognitive performance. As little as 0.5 mg/kg improves alertness, vigilance, and attention [4]. Doses of 1-3 mg/kg can decrease reaction time, increase speed, and improve memory [5]. Energy drinks are popular for their caffeine content in the e-gaming industry but can result in unwanted side effects including jitters, anxiety, and overarousal [5,6]. Due to the potential negative impact on gamer performance, it is beneficial to investigate a range of supplements that can improve cognitive performance.

Theacrine or 1,3,7,9-tetramethyluric acid is a compound that is similar to caffeine. It exerts its effects on adenosine and dopamine receptors resulting in a stimulant-like effect [7,8]. Studies report acute ingestion of theacrine results in improved cognitive function [7-10]. Of particular importance to e-gamers is its role in mood regulation and attention [7]. However, the effects of theanine did not continue to improve with chronic supplementation. Theacrine is generally considered safe and well-tolerated, with minimal side effects reported at typical doses making it an ideal candidate for supplementation in e-gamers [11]. Another ingredient of interest is methylliberine. This compound is structurally similar to methylxanthine caffeine and is thought to have similar physiological effects [12,13]. Notably, there have been a few that also report the cognitive benefits of methylliberine combined with theacrine [13].

The primary aim of this study was to evaluate the effects of combined caffeine + TeaCrine + Dynamine (CTD) measures of physiological changes and first-person shooter game performance in e-gamers. Based on previous studies utilizing similar ingredients [9,14,15], the researchers hypothesized that measures of gamer performance would improve following the acute ingestion of supplementation. Additionally, measures of mood, stress, and physiological arousal (salivary cortisol and EEG frequency bands) were assessed.

## Materials and methods

Experimental protocol

This study followed a randomized, double-blinded, crossover (counterbalanced for order effect), placebo-controlled design. Subjects reported to the clinical site on three separate visits with one week between each. All participants underwent an informed consent process in accordance with the Declaration of Helsinki and an approved IRB protocol submitted to the institutional review board of Keiser University (IRB000MR22JC112).

Subjects were instructed to arrive at the laboratory following their usual daily routine. All pre- and post-testing occurred between 12.00 and 17.00 hours. Testing sessions consisted of three visits that were one week apart. During the first visit, demographics, anthropometrics, and gaming history were assessed. At every test, each subject would complete the following protocol: vitals (blood pressure and resting heart rate), the profile of moods states (POMS), mood, alertness, and physical sensations scales (MAPSS), saliva sample collection, and complete AIMLAB (State Space Labs, Inc., New York, USA) games (speed, precision, and Standard). After gaming was completed, participants used a visual analog scale (VAS) to self-assess their gaming performance. Next, participants would consume supplements/placebo/caffeine. After 60 minutes, participants would repeat the following tests: vitals, POMS, MAPSS, saliva sample collection, and AIMLAB games. This was followed by a self-assessment of gamer performance and a third saliva sample. Participants also wore a brainwave-sensing headband (Enchanted Wave, LLC, Florida, USA) to record neurophysiological activity during gaming.

Subjects

All participants (n=49) were healthy (via self-report) adult men (mean age = 24.42 ± 4.52). Participants were amateur or semi-professional e-gamers who average at least 10 hours of gaming/week playing first-person shooter games. All participants were non-smokers and habitual caffeine consumers. Participants were excluded from the study if they were unhealthy, allergic to supplement ingredients, or had a history of mental health concerns 13 months prior to the study. Participants were instructed to maintain a stable lifestyle with no change in exercise, diet, or gaming for the duration of the study. Participants were also asked to refrain from caffeine consumption prior to testing on the day of testing.

Supplement

Subjects consumed a matched placebo, caffeine (200 mg), and caffeine (200 mg) + TeaCrine/Dynamine (CTD). The dietary supplement was provided by Compound Solutions™ (Carlsbad, California, USA). Both matched placebo, caffeine, and supplement were identical in size, shape, and color.

POMS

The POMS was used in the current study to assess acute mood. The POMS is a 65-item self-report psychological instrument intended for use with adults aged 18 and above and provides a composite score of total mood disturbance (TMD) [16].

MAPSS

The MAPSS includes 19 items describing moods and physical sensations [17]. The MAPSS includes three subscales based on alertness, anxiety, and headache.

Cortisol

Saliva samples were obtained from each participant via passive drool. The samples were collected in 1.5 mL polypropylene microcentrifuge tubes and kept on ice until the session was complete. Following the session, the samples were stored in a freezer at -20°C until assayed. Saliva samples were run in duplicates. Cortisol was quantified via a human enzyme immunoassay kit according to the manufacturer’s instructions (Salimetrics, Carlsbad, CA, USA). The functional sensitivity of the salivary cortisol enzyme-linked immunosorbent assay is 0.018 μg/dL.

Neurophysiological activity

EEGs were recorded by using a single-channel EEG (Enchanted Wave, LLC, South Florida, USA) with the electrode positioned at the prefrontal-lobe location (Fp1) based on the 10-20 system of electrode placement. At the end of each recording, the device’s automated analysis applies a fast Fourier transform to transform the raw EEG signal from the time domain into standard EEG frequency domains (alpha, beta, delta, theta).

Gaming performance (AIMLAB)

Gaming performance was assessed through a series of first-person shooter training games through AIMLAB. These included Reflex Shot (RS) standard, speed, and precision.

VAS: perceived performance

To assess subjective ratings of gamer performance, participants rated their perceived level of performance using a 10-point scale (i.e., 0 for poor performance and 10 for exceptional performance). They completed this assessment after each AIMLAB gaming session.

Statistical analysis

All calculations and analyses were performed using SPSS Statistics version 28.0.1 (IBM Corp. Released 2021. IBM SPSS Statistics for Windows, Version 28.0. Armonk, NY: IBM Corp.). Outliers on individual tasks were identified and removed using the interquartile range. For each measure, a repeated measures (RM) ANOVA was first conducted with time (pre- and post-dose) and session (placebo, caffeine, CTD) as within-subjects measures. These were followed up with planned comparisons based on our previous findings [14]. Paired samples t-tests were used to compare pre- and post-dose for each product condition. Post-dose scores were also compared between product conditions using paired samples t-tests. The significance level was at p <0.05, and all t-tests used a two-tailed p-value.

## Results

POMS

**Figure 1:**
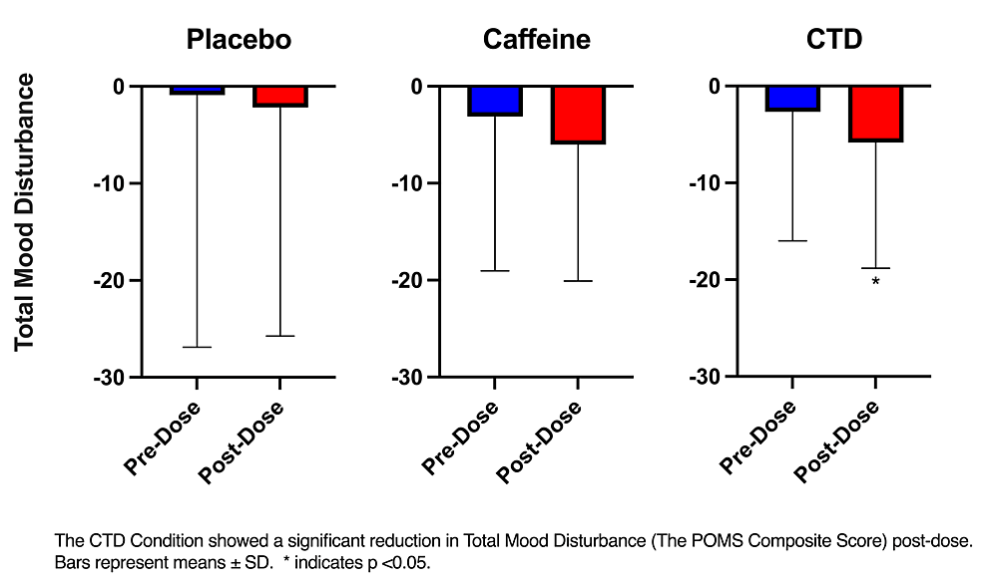
POMS TMD CTD: caffeine + TeaCrine + Dynamine, POMS: profile of moods states

The RM ANOVA (time x condition) only showed a significant effect of time in TMD (F(1, 35) = 7.45, p = 0.01). Post-hoc analyses showed a significant reduction in TMD in the CTD condition pre- vs post-dose (p=0.04) (Figure 1). Note that lower scores indicate less mood disturbance.

MAPSS

**Figure 2:**
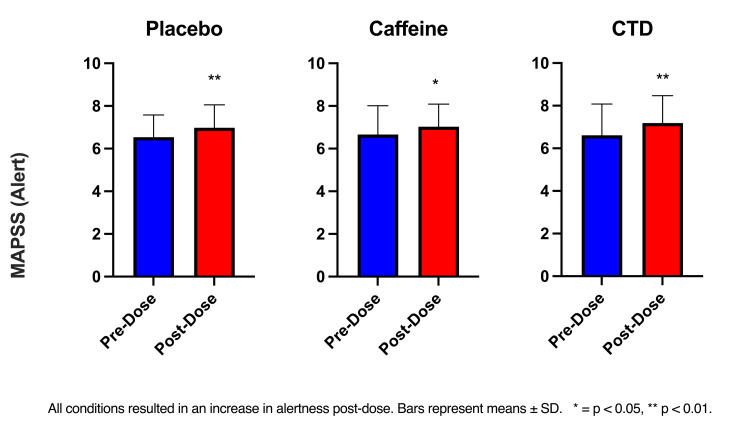
MAPSS alertness post-dose comparisons MAPSS: mood, alertness, and physical sensations scales, CTD: caffeine + TeaCrine + Dynamine

The RM ANOVA (time x condition) showed a significant effect of Time (F(1, 42) = 32.90, p <0.01) in the alertness subscale. All conditions significantly increased alertness pre- to post-dose (all p’s <0.05) (Figure 2). Compared to placebo, caffeine significantly improved alertness post-dose (p <0.01). The RM ANOVA (time x condition) showed a significant effect of condition (F(2, 72) = 6.35, p <0.01) in the anxiety subscale. Post-hoc tests showed that compared to the placebo, there was a significant increase in anxiety post-dose in the CTD (p <0.001) and caffeine (p <0.001). No significant changes were reported in the headache subscale.

Cortisol

**Figure 3:**
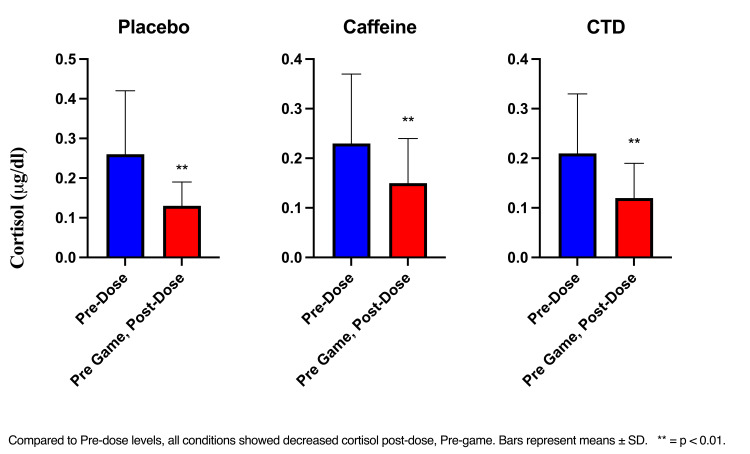
Cortisol pre-dose, post-dose, pre-game CTD: caffeine + TeaCrine + Dynamine

The RM ANOVA (time x condition) showed a significant effect of time (F(2, 70) = 36.35, p < 0.01) and a time x condition interaction (F(4, 140) = 4.31, p < 0.01). Post-hoc tests showed that compared to pre-dose levels, all conditions showed decreased cortisol post-dose, pre-game (all p’s <0.001) (Figure 3). Cortisol levels also significantly decreased in all conditions pre-dose compared to post-dose post-game (placebo and caffeine p <0.001, CTD p < 0.05). Between-group comparisons showed that compared to the placebo condition, cortisol levels were significantly higher in the placebo (<0.001) and CTD group (p = 0.01) post-dose, post-game.

Neurophysiological activity

**Figure 4:**
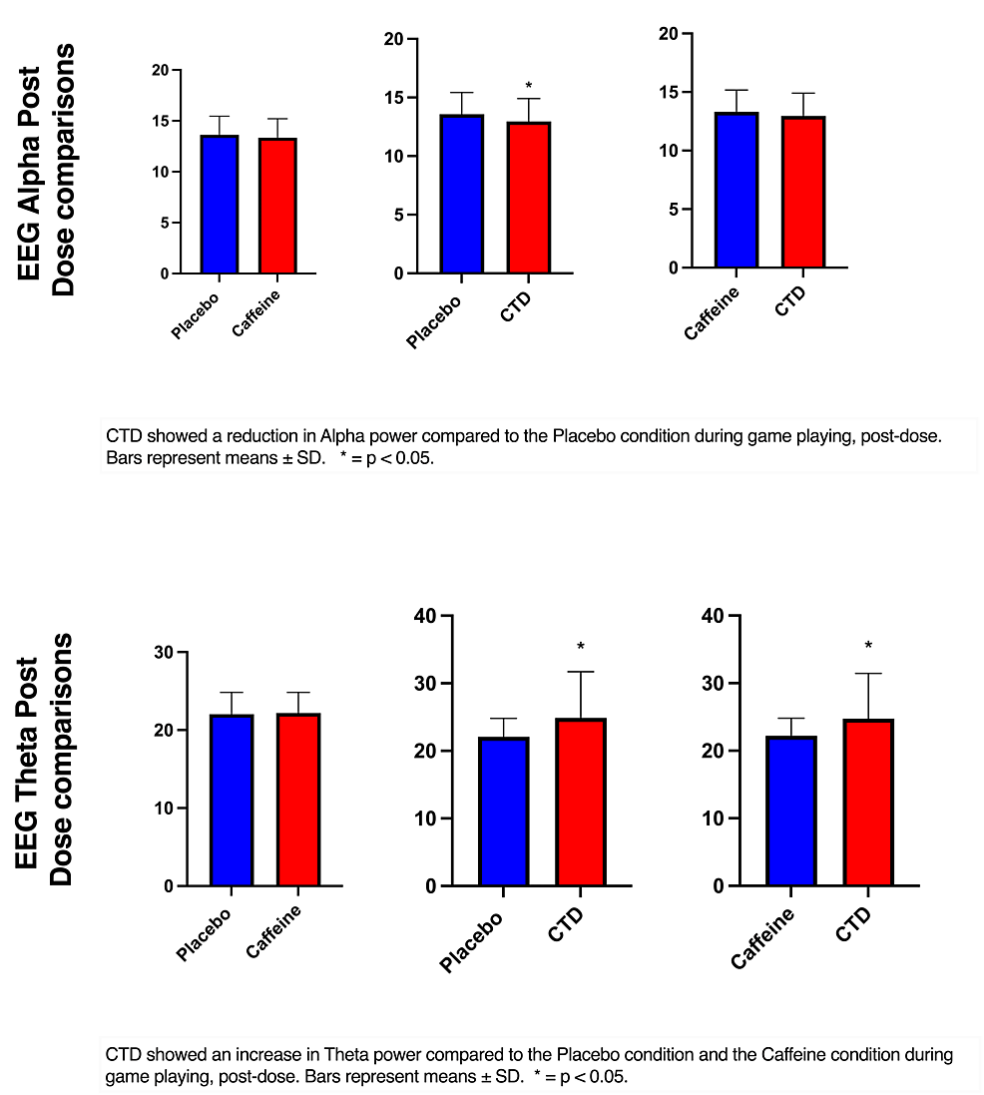
EEG post-dose comparisons CTD: caffeine + TeaCrine + Dynamine

The RM ANOVA (time x condition) showed a significant effect of condition (F(2, 86) = 4.62, p =0.01) in the alpha band frequency. Post-hoc analysis showed a reduction in alpha power compared to the placebo condition during game playing, post-dose (p=0.02) in the CTD condition (Figure 4). There was also an increase in theta power in the CTD condition compared to the placebo condition (p<0.001) and the caffeine condition (p<0.001) during game playing, post-dose condition (Figure 4).

Gaming performance (AIMLAB)

Standard

**Figure 5:**
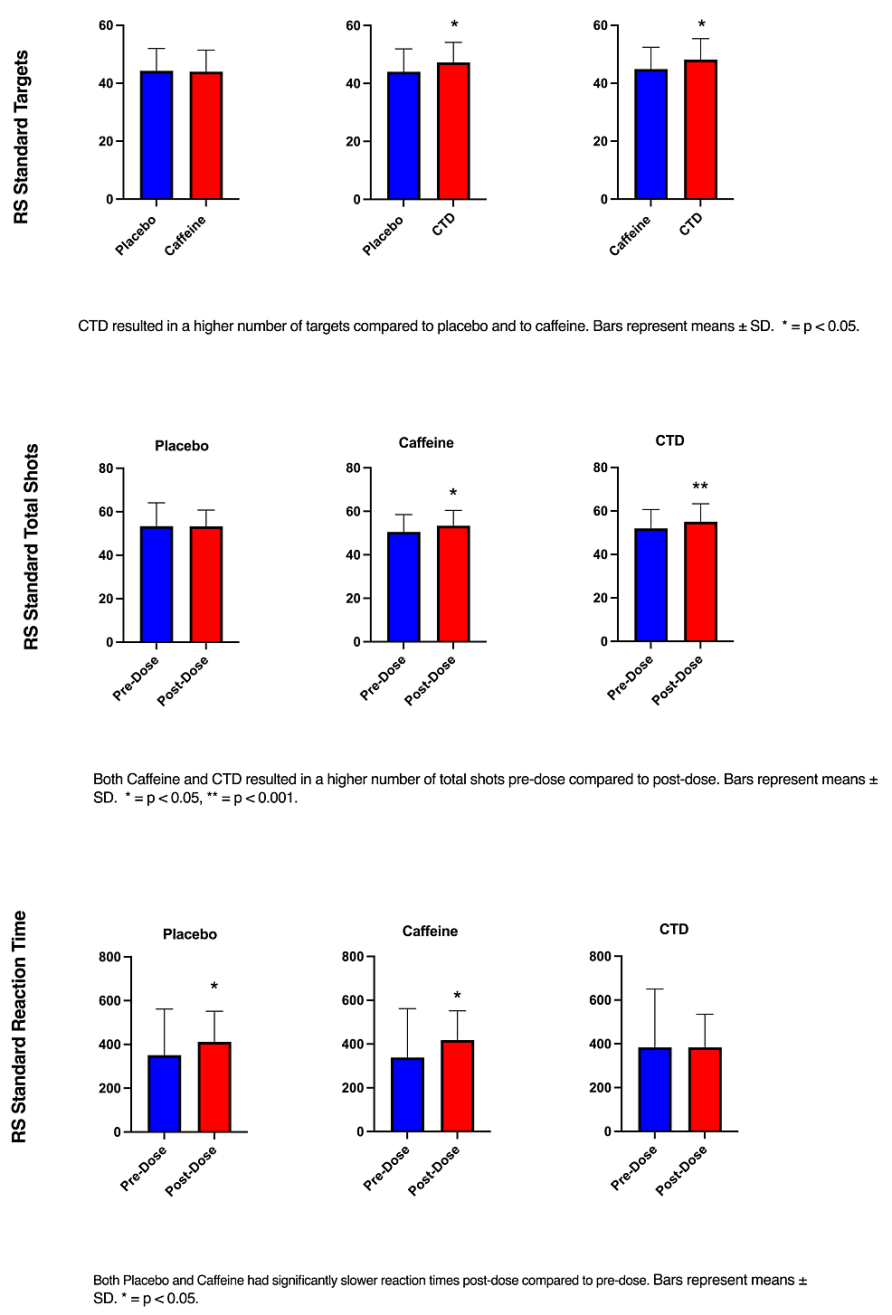
Standard gaming performance CTD: caffeine + TeaCrine + Dynamine

The RM ANOVA (time x condition) showed a significant effect of time (F(1, 25) = 49.24, p <0.001) for the number of targets hit. Post-hoc analysis revealed that all conditions increased the number of targets hit pre- to post-dose (P <0.001). The CTD condition hit significantly more targets compared to caffeine (p=0.01) and placebo (p=0.03) post-dose. There was also a significant improvement in the number of kills per second from pre- to post-dose in all conditions (p <0.001). The RM ANOVA (time x condition) showed a significant effect of Time (F(1, 25) = 7.13, p =0.01) in total shots. Post-hoc analysis showed that both caffeine (p=0.01) and CTD (p=0.008) significantly increased the number of shots taken. No significant differences in total kills were shown in any of the conditions (Figure 5).

Speed

**Figure 6:**
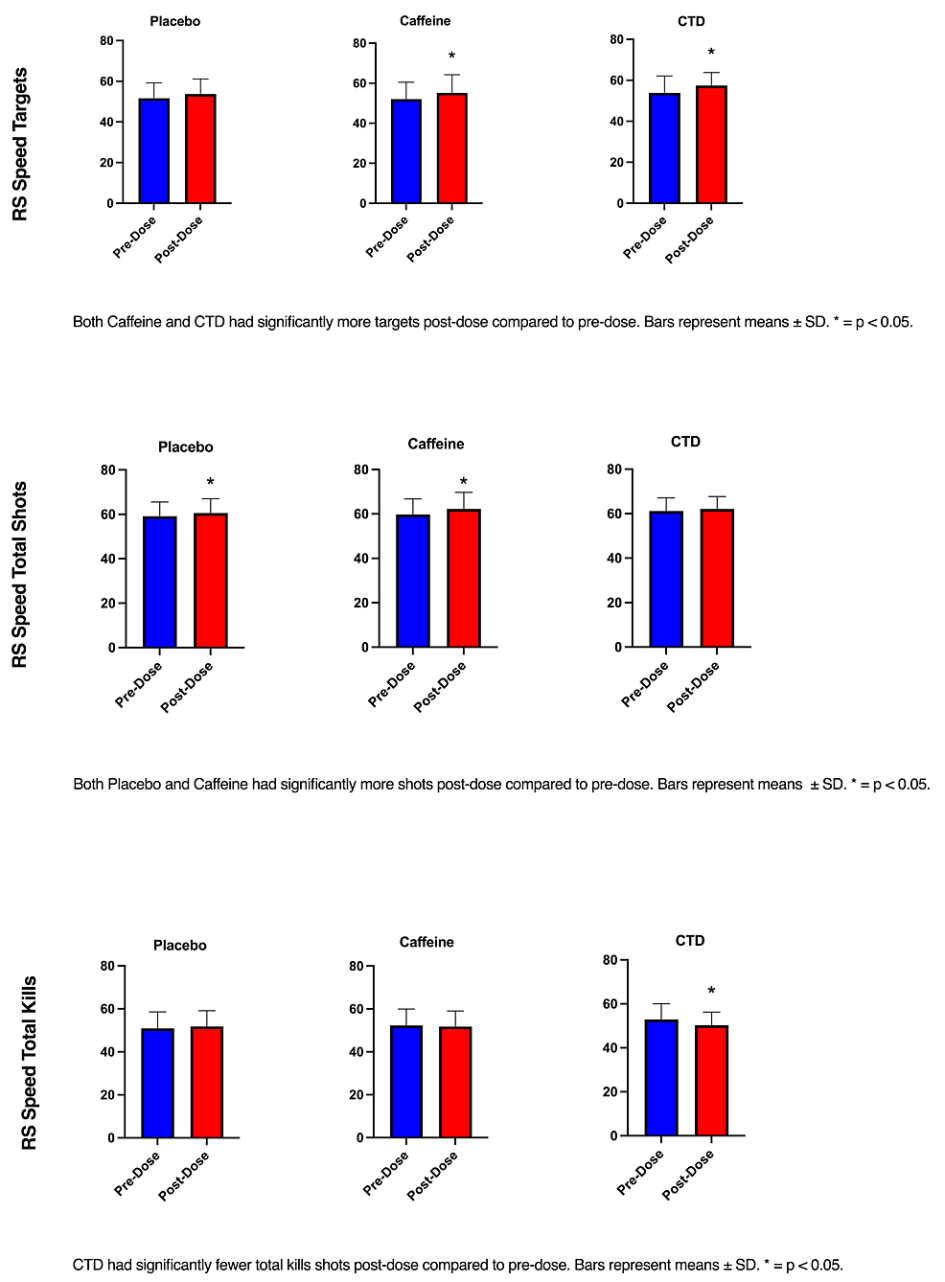
Speed gaming performance CTD: caffeine + TeaCrine + Dynamine

The RM ANOVA (time x condition) showed a significant effect of time (F(1, 22) = 18.21, p <0.001) for the number of targets hit. Post-hoc analysis showed a significant increase in the number of targets hit from pre- to post-dose in the caffeine condition (p=0.03) and CTD condition (p=0.004). Post-hoc analysis showed a significant increase in kills per second from pre- to post-dose in the placebo condition (p=0.04). The RM ANOVA (time x condition) showed a significant effect of time (F(1, 22) = 6.66, p =0.02) in the total shots taken. Post-hoc analysis showed a significant increase in total shots taken between pre- and post-dose in the placebo (p=0.02) and caffeine (p=0.01) conditions. Only the CTD condition significantly increased the number of total kills from pre- to post-dose (p=0.02) (Figure 6).

Precision

**Figure 7:**
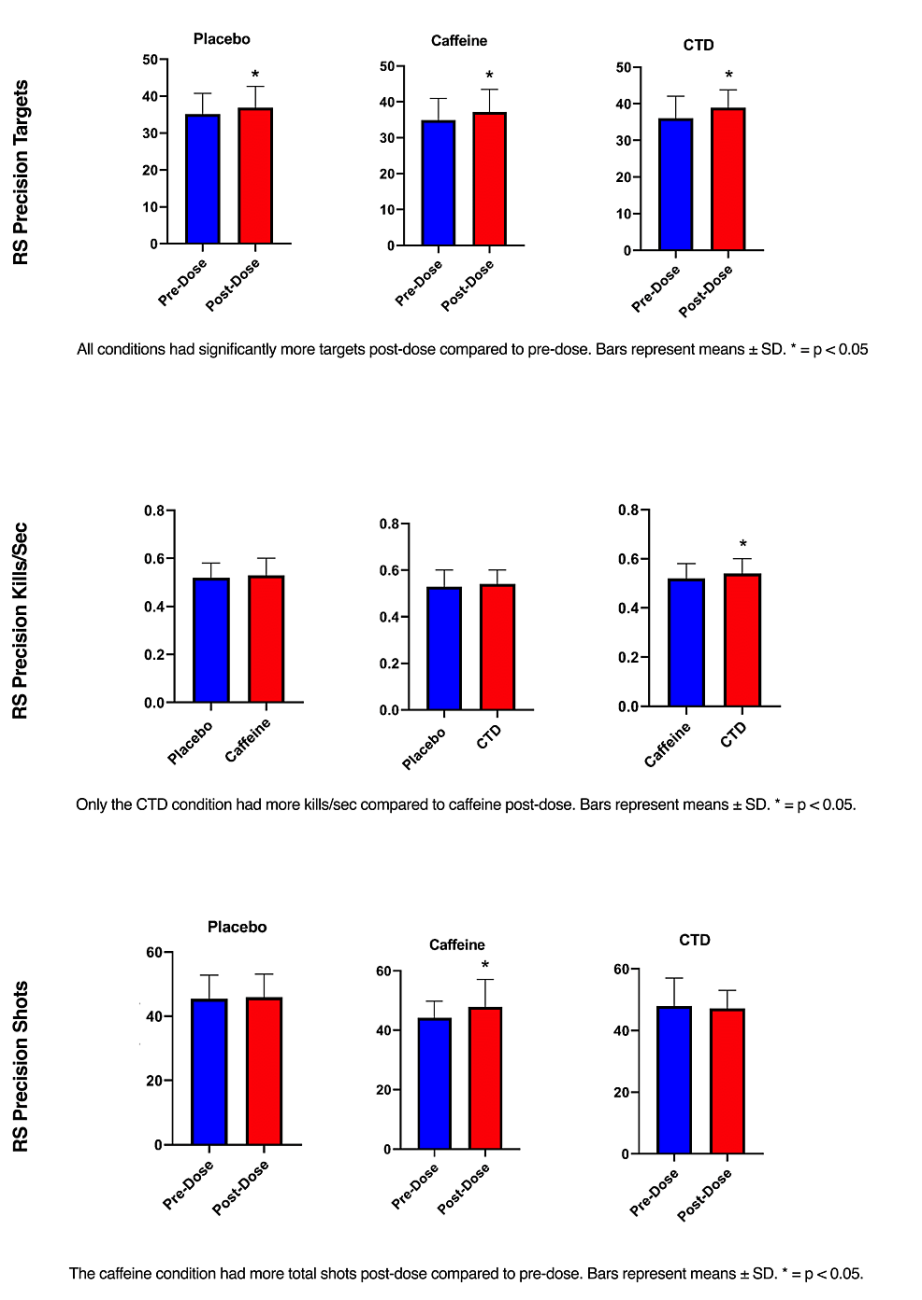
Precision gaming performance CTD: caffeine + TeaCrine + Dynamine

The RM ANOVA (time x condition) showed a significant effect of time (F(1, 24) = 11.70, p <= 0.00). Post-hoc analysis showed a significant improvement in the number of targets on the post-dose testing compared to the pre-dose testing (p <0.05) in all conditions. Only the CTD condition significantly improved on number of kills per second pre-dose compared to post-dose (p=0.01). The CTD condition showed significantly more kills/second compared to caffeine post-dose (p<0.05). Only the caffeine condition had significantly more shots post-dose compared to pre-dose (p=0.003) (Figure 7).

VAS: perceived performance

**Figure 8:**
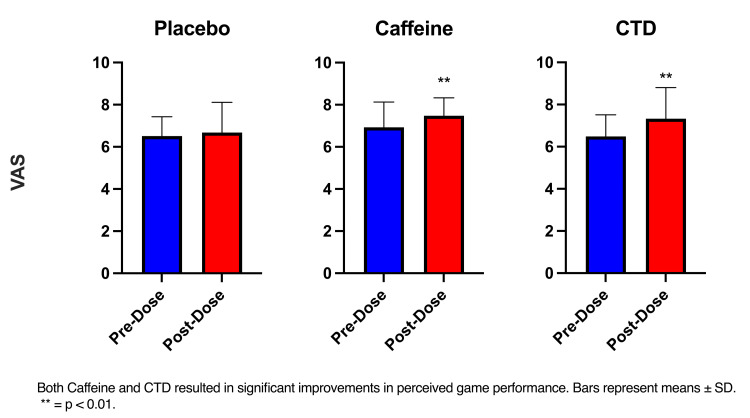
Visual analog scale pre-post CTD: caffeine + TeaCrine + Dynamine

The RM ANOVA (time x condition) only showed a significant effect of condition (F(2, 66) = 5.05, p < 0.01) and a main effect of time (F(1, 33) = 18.31, p < 0.01) Post-hoc analysis showed significant improvements in perceived performance post-dose and post-dose compared to pre-dose in caffeine and CTD (p <0.001) (Figure 8).

## Discussion

This study used a randomized double-blinded, crossover design to evaluate the effects of an acute dose of CTD, caffeine, and matched placebo in amateur, male e-gamers. We tested the effect of each condition on measures of neurophysiological activity, as well as performance, during first-person shooter games in e-gamers.

The stimulant effect of caffeine is thought to increase feelings of vigor and/or decrease feelings of fatigue. No changes in vigor, tension, or anxiety side effects commonly attributed to caffeine were reported in this study. All participants were habitual caffeine users which may influence their response to caffeine. When combined with caffeine, theacrine may offset some of the negative side effects of caffeine which may explain the significant reduction in total mood disturbance reported in the CTD group only [18]. However, it is worth noting that levels of anxiety via MAPSS were not significantly different between the caffeine and CTD conditions. These findings differ slightly from research examining the effects of caffeine and theacrine. Xu et el. (2007) [18] found theacrine to offset caffeine-induced anxiety in mice. Tartar et al. [14] reported an increase in alertness but not anxiety following the ingestion of a CTD supplement. It is possible that the present results differ due to the nature of the assessments used. The current study used a first-person shooting assessment, while the previous study used standard cognitive tasks. All conditions experienced an increase in perceived alertness via MAPSS pre-post dose. The increase in the placebo group may be the result of the “placebo effect” which tends to be higher on self-reported outcomes such as those used in MAPSS [19].

The higher cortisol levels observed at baseline suggest greater physiological arousal. This can be attributed to anticipatory arousal since the participants were mentally preparing for the upcoming gaming assessments. In agreement with this idea, there were significant decreases that occurred at the post-dose, post-game timepoint in all conditions. However, the CTD and caffeine conditions had higher cortisol compared to the placebo condition. This may indicate participants entering the flow state. The inverted U-shape theory supports the relationship between arousal and performance [20]. Under- or overarousal negatively impacts performance. However, an optimal amount of arousal allows individuals to get into a flow state and enhances performance [20,21]. These findings are in agreement with those reported by Tartar et al. [14] where cortisol was higher in the supplement and caffeine condition compared to the placebo. The authors suggest increases in energy mobilization as a possible cause of higher cortisol levels [14].

EEG analyses showed that compared to the matched placebo, alpha power was lower in the CTD condition. Decreases in alpha power are typically observed during visual processing when switching between tasks is necessary and are correlated with improved short-term memory [22,23]. These results suggest cognitive flexibility or the ability to shift attention between tasks which is necessary for first-person shooting [24]. Following CTD supplementation, theta power was significantly higher compared to matched placebo and caffeine. Increases in theta power are indicative of increased executive control [25]. It has been suggested that higher theta activity minimizes distracting activity such as those experienced during first-person shooter games [26]. The reported increase in theta power is in line with previous research using CDT [14].

During the RS standard games, both caffeine and CTD conditions took significantly more shots and hit significantly more targets during the post-dose assessment. However, both placebo and caffeine had significantly slower reaction times during post-dose game playing. This suggests a greater speed-accuracy trade-off in the placebo and caffeine conditions compared to CTD since CTD resulted in participants responding faster without sacrificing accuracy. These findings also support the notion that caffeine is an effective ergogenic aid for e-sport gamers [5,6,27]. During the RS speed game, caffeine and CTD conditions hit significantly more targets during game playing post-dose. Only the placebo group increased the number of total kills post-dose gaming. Interestingly, the placebo and caffeine groups took significantly more shots compared to the CTD group. This may be the result of the so-called spray-and-pray approach and aligned with those of La Monica et al. [27]. Although this study used different games on the AIMLAB platform, they reported faster reaction times and a higher degree of precision following supplementation with CTD. Caffeine is known for its ability to increase reaction time but sometimes at the cost of accuracy/precision [5,6,27]. The addition of TeaCrine appears to enhance caffeine’s effects while minimizing the negative effects, as evidenced by our findings. The results of gaming in RS precision support the notion that CTD enhances accuracy in this study. Despite all conditions (matched placebo, caffeine, and CTD) increasing the number of targets hit following supplementation, only the CTD group resulted in a greater number of kills per second pre- to post-dose. These results strongly support the synergistic effects of CTD in first-person shooter games.

Subjective assessments of gaming performance provide important insight into an e-gamers perceived mental effort and task difficulty. Previous studies report increased subjective feelings of motivation, focus, and attention following supplementation with a combination of CTD [9,10,15,27]. Our findings are in line with La Monica et al. [27] where CTD supplementation improved subjective ratings of performance in first-person shooter video games and improvements in their gaming performance compared to the caffeine group [27]. It appears that CTD is effective at enhancing an individual’s desire to perform and perceived rating of performance in both video games and other cognitively demanding tasks.

The primary limitation of this study is the use of a mouse rather than a handheld game controller when playing video games. To overcome this challenge, participants were instructed to play multiple rounds for each game during every condition. We also employed a randomized repeated measures design to ameliorate this concern. An additional limitation is the use of only male participants. Future studies are needed to see if these results apply to females as well.

## Conclusions

CTD appears to improve overall shooting gaming performance and neurophysiological measures of cognitive activity while also providing general mood benefits relative to caffeine and placebo. CTD also appears to benefit overall mood as there was a significant decrease in mood disturbance in the CTD condition, but not the placebo or caffeine conditions. During the standard shooting game, both caffeine and CTD groups took significantly more shots and hit significantly more targets; however, only the CTD condition did not slow down reaction time. In the speed game, CTD supplementation resulted in fewer total kills, while caffeine and placebo conditions took more shots. In the precision game, all conditions resulted in more shots taken, but only the CTD condition had significantly more kills per second. Combined, these findings suggest that CTD improves speed without sacrificing accuracy, which is particularly beneficial for shooting precision. The EEG data support this notion since the CTD condition exhibited higher alpha power suggesting increase cognitive flexibility and lower theta power suggesting greater cognitive control. Combined, the data from this study show that CTD possibly improves gaming performance through benefits to neurophysiological and mood measures.
